# Functional and Molecular Characterization of Plant Nitrate Transporters Belonging to NPF (NRT1/PTR) 6 Subfamily

**DOI:** 10.3390/ijms252413648

**Published:** 2024-12-20

**Authors:** Olga I. Nedelyaeva, Dmitry E. Khramov, Yurii V. Balnokin, Vadim S. Volkov

**Affiliations:** K.A. Timiryazev Institute of Plant Physiology RAS, 127276 Moscow, Russia; khramov.de@yandex.ru (D.E.K.); balnokin@mail.ru (Y.V.B.)

**Keywords:** NPF6 subfamily, transporter, nitrate, halophyte, proton-coupled oligopeptide transporters, salinity

## Abstract

Plant nitrate transporters in the NPF (NRT1) family are characterized by multifunctionality and their involvement in a number of physiological processes. The proteins in this family have been identified in many monocotyledonous and dicotyledonous species: a bioinformatic analysis predicts from 20 to 139 members in the plant genomes sequenced so far, including mosses. Plant NPFs are phylogenetically related to proton-coupled oligopeptide transporters, which are evolutionally conserved in all kingdoms of life apart from Archaea. The phylogenetic analysis of the plant NPF family is based on the amino acid sequences present in databases; an analysis identified a separate NPF6 clade (subfamily) with the first plant nitrate transporters studied at the molecular level. The available information proves that proteins of the NPF6 clade play key roles not only in the supply of nitrate and its allocation within different parts of plants but also in the transport of chloride, amino acids, ammonium, and plant hormones such as auxins and ABA. Moreover, members of the NPF6 family participate in the perception of nitrate and ammonium, signaling, plant responses to different abiotic stresses, and the development of tolerance to these stresses and contribute to the structure of the root–soil microbiome composition. The available information allows us to conclude that *NPF6* genes are among the promising targets for engineering/editing to increase the productivity of crops and their tolerance to stresses. The present review summarizes the available published data and our own results on members of the NPF6 clade of nitrate transporters, especially under salinity; we outline their molecular, structural, and functional characteristics and suggest potential lines for future research.

## 1. Introduction

Nitrogen is one of the most important macro-nutrients for plants. Nitrogen deficiency leads to the inhibition of plant growth and reduces crop yields. The largest share of nitrogen enters plant roots in the form of nitrate. The concentration of nitrate in a soil solution depends on many factors and varies over a wide range, from a few µM to several tens of mM [1,2]. Physiological experiments indicate that nitrate is taken up by plant roots via specific transport systems. Based on their affinity for nitrate, transporters involved in nitrate uptake are divided into low-affinity transport systems (LATS) and high-affinity transport systems (HATS). Low-affinity transport systems are defined as having a Km greater than 0.5 mM, while they often do not saturate above 50 mM (reviewed in [3]). HATS are characterized by Km values below 0.5 mM down to 6–11 μM (reviewed in [3]; e.g., [4,5,6,7]). Among these low-and high-affinity nitrate transporters, there are constitutively expressed (cLATS and cHATS) and inducible systems (iLATS and iHATS) [2,4,8,9,10,11,12,13].

At the molecular level, four families of protein channels and transporters are involved in nitrate uptake, allocation among different parts of a plant, and storage in vacuoles: NRT1 (NPF/PTR) (Nitrate Transporter 1/Peptide Transporter Family) (nomenclature in: [12]), NRT2 (Nitrate Transporter 2) [14,15], CLC (Chloride Channel) [16,17,18] and SLAC/SLAH (Slow Anion-Associated Channel Homolog) [19,20]. Members of the NPF and NRT2 families are responsible for nitrate uptake by plant cells, transporting nitrate in symport with protons with a H^+^:NO_3_^−^ stoichiometry over 1:1, typically 2:1. The energy source for the nitrate uptake is its electrochemical potential gradient, which is linked to the activity of the plasma membrane H^+^-ATPase.

Nitrate transporters in the NPF family play important roles in anion uptake and transport within plants. This family includes from 20 to 139 members in the genomes sequenced so far for different plant and moss species [12,21]. Plant NPFs are phylogenetically related to proton-coupled oligopeptide transporters, which are evolutionally conserved in all kingdoms of life apart from Archaea (reviewed in [22]). These oligopeptide transporters and a wider group of proton-coupled nutrient transporters include transporters of di- and tripeptides (SLC15 family), some vitamins (SLC19 family), iron (SLC40) and others (reviewed in [22]); they are a part of a larger group of the Major Facilitator Superfamily of solute secondary transporters [23]. The proteins in the plant NPF family have been identified and studied or predicted based on bioinformatic data in *Arabidopsis thaliana* [12,24], *Brachypodium distachyon* [25], *Lotus japonicus* [26], *Sorghum bicolor* [27], *Medicago truncatula* [21,28,29,30], *Oryza sativa* [21], *Glycine soja* [31], *Solanum tuberosum* [32], *Spirodela polyrhiza* [33]; *Poncirus trifoliate* [34], *Triticum aestivum* [35,36], *Zea mays* [6,37], *Suaeda altissima* [38], *Zostera marina* [7], *Eutrema halophilum* [39], and many other plant species. Members of this family are involved not only in the transport of nitrate and chloride anions but also in transport of amino acids, di- and tripeptides, glucosinolates, dicarboxylic acids, and plant hormones (gibberellins, auxins, and ABA) [13,40,41].

**Figure 1:**
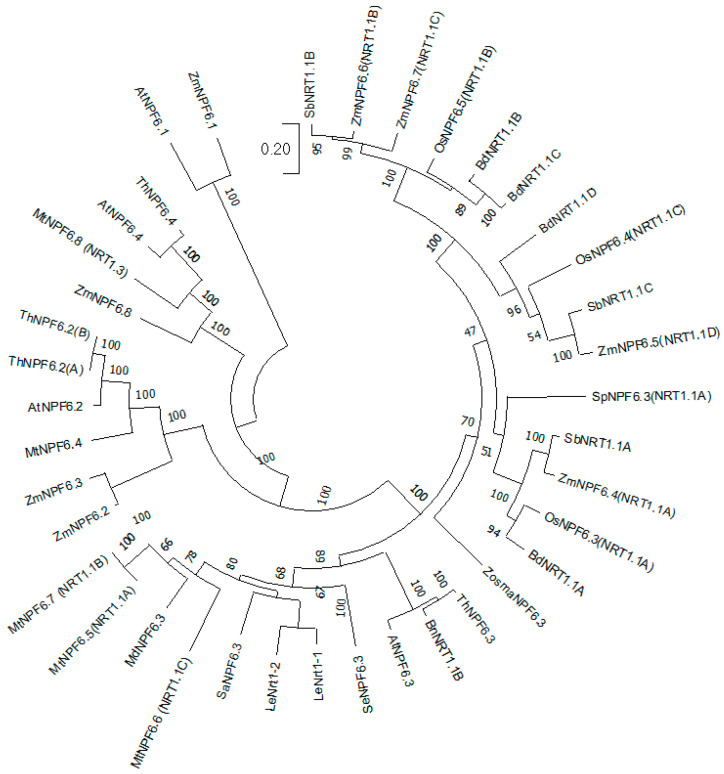
Phylogenetic analysis of the amino acid sequences of clade 6 proteins of the nitrate transporter NPF/NRT1 family. The phylogenetic tree was built in the program MEGA X v. 10.0.1 (https://www.megasoftware.net/ (accessed on 15 December 2024)) by the maximum likelihood method based on the Jones–Taylor–Thornton model. The results of the bootstrap analysis are given in nodes of the phylogram (1000 iterations); scale bar—0.20 replacements per site. Amino acid sequences of the proteins of *A. thaliana*, *M. truncatula*, *B. napus*, *L. esculentum*, *M. domestica*, *S. altissima*, *T. halophila*, *Z. mays*, *O. sativa*, *S. bicolor*, *B. distachyon*, *Z. mays*, *O. sativa*, *B. distachyon*, *Z. marina* for the phylogenetic tree construction were taken from NCBI; amino acid sequences of *S. polyrhiza*, *T. halophila*, and *S. europaea* proteins were taken from the Phytozome (https://phytozome-next.jgi.doe.gov/info/Spolyrhiza_v2 (accessed on 15 December 2024)) and *Salicornia* DB (https://www.salicorniadb.org/ (accessed on 15 December 2024)) databases. GenBank sequence numbers are given in the Supplementary Materials (Table S1).

So far, the best known phylogenetic analysis of the plant NPF family was based on 2398 amino acid sequences in the databases with members of both monocotyledonous and dicotyledonous plants; eight distinct unambiguous clades were identified [12]. The separate NPF6 clade is unique in the way that it includes the first discovered and the best studied plant nitrate transporters at the molecular level ([42]; Figure 1; Supplementary Materials Table S1, Figure S1). The available information suggests that proteins in the NPF6 clade are among the key players in the supply of nitrogen to plants; they influence the efficiency of nitrogen utilization during crop development. Therefore, *NPF6* genes are promising candidates for genetic engineering/editing to increase the yield and nitrogen use efficiency of crops. This review highlights the available data on members of the NPF6 clade of nitrate transporters and outlines their molecular, structural, and functional characteristics. The main focus is on the roles of NPF6 transporters under salinity and in halophyte plants.

## 2. Molecular Characteristics of Plant Transporters Belonging to the NPF6 Subfamily

**Figure 2:**
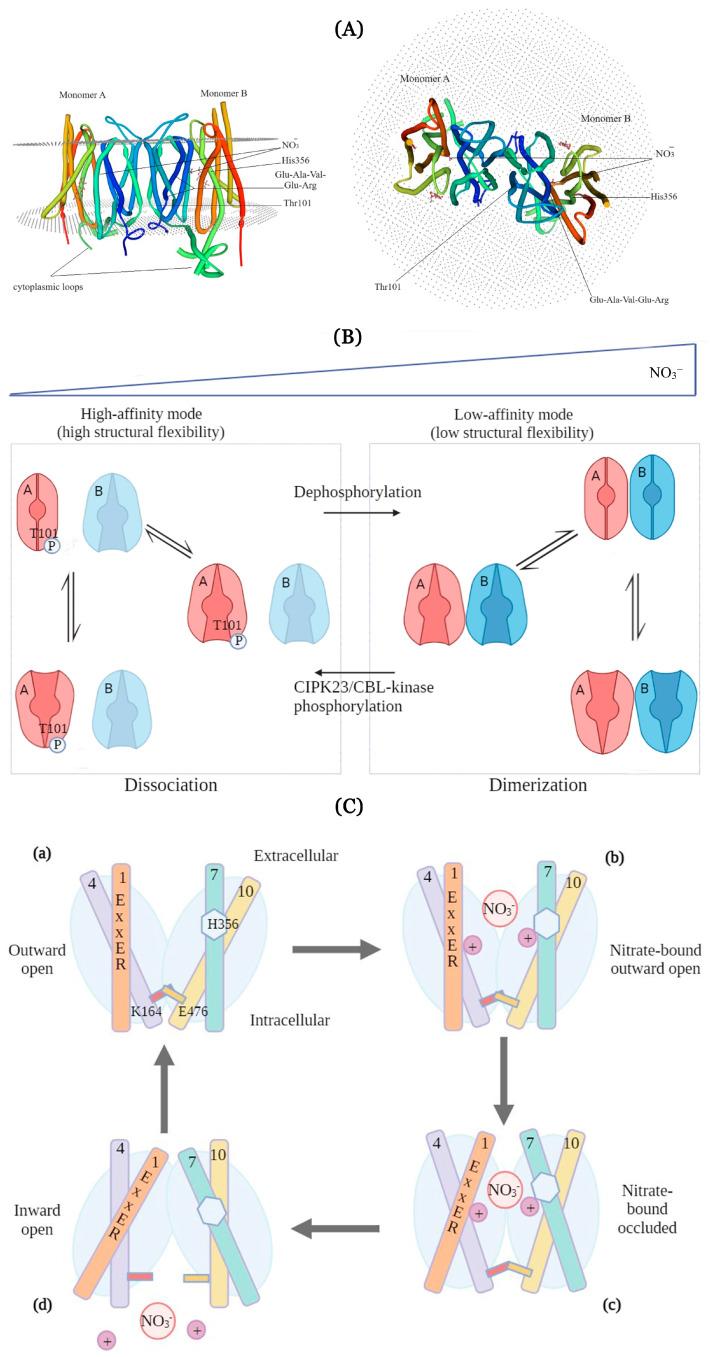
The dual-affinity nitrate transporter AtNPF6.3 of *Arabidopsis thaliana*. (**A**) The three-dimensional structure of the AtNPF6.3 dimer plotted in the Swiss-model program (https://swissmodel.expasy.org/ (accessed on 15 December 2024)) based on the crystal structure of this protein [44]. Transmembrane domains are colored differently. Structure of the AtNPF6.3 dimer composed of A and B protomers in the presence of NaNO_3_. Nitrate ions, the key amino acid residues and motifs, namely, the nitrate-binding His356, affinity mode-switching Thr101, and proton-binding ExxER, as well the cytoplasmic loops and the N-(TM1-TM6) and C-(TM7-TM12)-terminal domains including 6 α-helices each are indicated in both monomers. On the left is the side view, on the right is the top view. (**B**) Transition between the AtNPF6.3 transporter dimer composed of monomers A and B with low conformational mobility and monomer with high conformational mobility, which determine the low-affinity and high-affinity modes of nitrate binding, respectively. (**C**) Transport cycle of the AtNPF6.3 symporter. (a) Between amino acid residues K164 (atTM4) and E476 (at TM10), an ionic bond (salt bridge) is formed, which closes the pore (tunnel) and brings together the N-(TM1-6) and C-(TM7-TM12)-terminal domains of the subunit (outward-facing conformation). (b) After protonation of ExxER (at TM1) and H356 (at TM7), nitrate binding to H356 occurs. (c) The transporter switches to a closed (occluded) conformation, (d) the ionic bond (salt bridge) between K164 and E476 is broken, and the transition into the inward-facing conformation of the protein and the release of nitrate and two protons occurs.

The molecular structure of a representative of the NPF6 subfamily proteins is best studied for AtNPF6.3. In two independent investigations, the AtNPF6.3 protein was crystallized and its 3D structure was explored [43,44]. The AtNPF6.3 protein is a dimer consisting of two nearly identical A and B protomers: each formed by twelve transmembrane α-helices and cytoplasmic loops located at the N- and C-termini (TMH1-6 and TMH7-12, respectively) (Figure 2 depicts the results of modeling). The conserved Thr101 (TMH3) phosphorylation site at the N-terminus is completely immersed in a hydrophobic ‘pocket’ located at the contact site of the two monomers and formed by the amino acid residues of the transmembrane TMH2, TMH3, and TMH4 domains. The Thr101Asp and Thr101Ala amino acid substitutions, mimicking constitutive phosphorylation and dephosphorylation, switch the transporter between the high-affinity and low-affinity modes of nitrate transport, respectively [44,45,46]. At millimolar nitrate concentrations in the medium, when AtNPF6.3 functions as a low-affinity transporter, the two monomers are in the associated state. At micromolar concentrations, nitrate binds only to monomer A, initiating the phosphorylation of Thr101 of this monomer by the protein kinase CBL-INTERACTING PROTEIN KINASE 23 (CIPK23), which in turn leads to the dissociation of the dimer and transition of the transporter into the high-affinity operation mode involving only monomer A (Figure 2). Phosphorylation of Thr101 leads to an increase in the structural flexibility (conformational mobility) of the carrier polypeptide chain and a 2.8-fold increase in the rate of nitrate uptake [43]. At millimolar nitrate concentrations in the medium, the binding of nitrate by both monomers favors the retention of AtNPF6.3 in the dimer form, providing the low-affinity operation mode (Figure 2). This mechanism ensures that plants respond quickly to a sharp decrease in the soil nitrate concentration. Plants adapt to nitrate deficiency even before the induction of the gene expression of the NRT2 family of high-affinity nitrate transporters and their NAR family partners [43,44].

Resolving the three-dimensional structure of the AtNPF6.3 protein allowed the prediction of the transport mechanism model by which AtNPF6.3 symports nitrate with protons with a suggested 2 H^+^/1 NO_3_^−^ stoichiometry [43,44]. This model is based on a suggestion that a sequential transition of the protein between several conformational states occurs in which nitrate-binding His356 is orientated either to the extracellular environment or to the cytosol. When passing through the AtNPF6.3 tunnel, nitrate interacts sequentially with several sites that play important roles in the transport process. These sites include (from the outside of the membrane to the inside) the conserved ExxER motif (a.a.41–45, TMH1), amino acid residue His356 (TMH7), and a pair of residues, K164 (TMH4) and E476 (TMH10). ExxER and His356 are involved in nitrate binding and coupling nitrate transport to proton transport. The first step of the cycle involves the protonation of ExxER and His356 by two environmental protons, with K164 and E476 forming a salt bridge near the inner side of the membrane, which closes the nitrate pathway into the cytosol. Subsequent binding of nitrate to the protonated form of His356 leads to a protein conformational rearrangement causing the breakage of the salt bridge bond and opening the way for nitrate to enter the cytosol. In the last step of the cycle, after the separation of the two protons and nitrate from the complex, AtNPF6.3 acquires its original conformation. The importance of the ExxER/K motif and the salt bridge are indicated by the fact that any of the point substitutions Glu41Ala, Glu44Ala, Arg45Ala, and Lys164Ala disrupt the transport function of the carrier. The amino acid residue His356 (TMH7) is localized in the nitrate-binding pocket formed predominantly by hydrophobic amino acids, Leu49 (TMH1), Leu78 (TMH2), Val53 (TMH4), and Phe511 (TMH11), and the conformation of its side chain is coordinated by the nearby Tyr388 (TMH8) and Glu476 (TMH10) residues [44]. The His356Ala point substitution resulted in a complete cessation of nitrate transport by the carrier [42,44]. Nitrate binding with the protein induces conformational changes in the regions of His356 and Thr101, leading to structural asymmetry of the dimer and a 5-fold higher affinity for nitrate of monomer A over that of monomer B [47,48]. These structural differences in the two monomers are probably responsible for differences in their functions.

The conserved Pro492, located in the short loop between TMH10 and TMH11, has also been shown to be essential for the transport function of the protein. The Pro492Leu point substitution mutant *atchl1-9* was defective in transport function, but its sensory function (see below) was retained [49]. The AtNPF6.3 protein has three conserved hydrophilic regions: (1) the extracellular short loop, which is located between the TM3 and TM4 domains and stabilized by the disulfide bond formed by the Cys130 and Cys137 residues; (2) the central helix between TM6 and TM7; and (3) the N-terminal domain facing the cytosol and likely carrying major binding sites for regulatory proteins [44].

## 3. Substrate Specificity of NPF6 Subfamily Transporters with the Functional Roles Involved

### 3.1. Transport of Nitrate and Chloride with Physiological Implications

A large number of plant NPF6 family transporters have been cloned and their expression profiles and transport properties have been studied (Table 1). The transport of nitrate and chloride by NPF6 subfamily transporters from different plants was shown directly or indirectly by several independent methods, including anion transport kinetics, gene expression analysis, heterologous expression in frog oocytes and in yeast cells, and by rescuing mutant phenotypes defective in nitrate transport.

The *A. thaliana* AtNPF6.3/AtNRT1.1 transporter was the first nitrate transporter discovered in plants; it was originally named CHL1 (chlorate resistant 1). The *AtNPF6.3* gene was identified by screening the T-DNA library of *Arabidopsis thaliana* mutants resistant to chlorate, a toxic structural analogue of nitrate [24]. The contribution of AtNPF6.3 to the total nitrate taken up by plants ranges from 10 to 80%, depending on the nitrate concentration in the soil solution [4,63,64]. Despite AtNPF6.3 being attributed to the family of low-affinity nitrate transporters, this protein, unlike most other *A. thaliana* NPF members, has dual affinity for nitrate. AtNPF6.3 is able to transport nitrate in both low-affinity (in the millimolar range of external nitrate concentrations) and high-affinity (at micromolar concentrations) modes, exhibiting biphasic saturating nitrate uptake kinetics with Km ≈ 4 mM and 40 to 80 μM, respectively [4]. The transition between these two states is regulated by changing the phosphorylation status of a specific Thr101 residue [45,65]. Using the *Xenopus* oocyte expression system, AtNPF6.3 has been shown to transport nitrate (reviewed in [42]). The *AtNPF6.3* gene is expressed in all organs and tissues of *A. thaliana*, but the highest transcript level was found in roots [66,67]. Its expression is induced by nitrate and inhibited by reduced nitrogen compounds, such as ammonium and nitrite [66]. Nitrate deficiency resulted in lowering the *AtNPF6.3* transcript level in roots [66,68] and the induction of its expression in leaves [15,69]. The AtNPF6.3 protein is localized in root cell plasma membranes of epidermal and conductive tissues, indicating its involvement not only in nitrate uptake but also in loading nitrate into the xylem [63,70]. AtNPF6.3 was also found in guard cells, suggesting its involvement in the regulation of stomatal conductance [71]. The genome-wide study of *Arabidopsis* accessions with various sensitivities to nitrogen deficiency demonstrated that enhanced *NRT1.1* expression in shoots facilitates nitrogen assimilation and plant growth under N-deficient conditions [69]. Apart from AtNPF6.3, three more members of the NPF6 subfamily are known in *A. thaliana*, namely, AtNPF6.1, AtNPF6.2 (NRT1.4), and AtNPF6.4 (NRT1.3) (Figure 1); the latter two have been partially characterized. The AtNPF6.2 protein is a plasma membrane nitrate transporter expressed predominantly in leaf petioles and carrying out low-affinity nitrate transport [51]. In the knockout mutant of the *AtNPF6.2* gene, *atnrt1.4*, the nitrate content in petioles was reduced almost 2-fold compared with wild-type plants, and nitrate was translocated into the leaves, which were wider compared to wild type [51]. The *A. thaliana* protein AtNPF6.4 is expressed in flowers, the stem cortex, and leaf mesophyll cells. The expression of AtNPF6.4 was induced in shoots by nitrate addition to the medium [52].

NPF6 nitrate transporters were identified and well studied in the agriculturally and economically important crop rice. The rice nitrate transporters OsNPF6.3 (OsNRT1.1A) and OsNPF6.5 (NRT1.1B) were identified using the *Xenopus* oocyte expression system, ^15^N-labelled nitrate, and mutants for genes encoding these proteins [61,62]. Oocytes expressing both *OsNPF6.3* and *OsNPF6.5* genes demonstrated low- and high-affinity nitrate transport activity, taking up nitrate from the media with nitrate concentrations of 10 mM and 200 µM. The *OsNPF6.3* and *OsNPF6.5* genes were expressed predominantly in the root epidermis and vascular tissues, revealing similar expression patterns; fewer OsNPF6.3 transcripts were found in stem and leaf parenchyma [61,62,72]. Among all known AtNPF6.3 homologs of rice, OsNPF6.3 shares the greatest similarity in amino acid sequence to AtNPF6.3; however, the rice transporter differs from the *Arabidopsis* protein and other previously characterized NPF6.3s in several features. Firstly, unlike AtNPF6.3 and all other NPF6.3s, OsNPF6.3 was found to localize in vacuolar membranes. This implies the involvement of OsNPF6.3 in nitrate storage in vacuoles and nitrate efflux from vacuoles when the intracellular N status alternates following the fluctuation of nitrogen availability in the environment. Secondly, *OsNPF6.3* expression is induced by ammonium, suggesting that this transporter is involved in ammonium utilization, which is the most available form of nitrogen for rice under flooded conditions [61]. Nitrate and ammonium uptake experiments with *OsNPF6.3* knockout mutants, different nitrogen sources, and hypoxia are required to answer the question of OsNPF6.3 multifunctionality.

Nitrate transport activity has also been shown for cloned NPF6 transporters from maize [6,42], see below for more details. By similarity to *AtNPF6.3*, a cDNA for a membrane transporter BnNRT1.2/BnNRT1B was isolated from *Brassica napus*. Using the *Xenopus* oocyte expression system, BnNRT1.2 was shown to be a low-affinity nitrate transporter [57]. The transport of nitrate displayed a dependence on a proton gradient, which suggests a nitrate–proton cotransport mechanism. The affinity of the transporter for nitrate was dependent on the membrane potential. The Km for nitrate increased from 4 to 14 mM as the membrane potential decreased from −40 to −180 mV [57]. Two genes homologous to *AtNPF6.3*, *LeNRT1-1* and *LeNRT1-2*, were detected in a cDNA library derived from the total RNA from tomato *Solanum lycopersicum* (*Lycopersicon esculentum*) root hairs [58]. The expression of both genes was organ-specific; their transcripts were found only in root tissues and undetectable in tomato leaves and stems. *LeNRT1-1* was constitutively expressed in all root tissues with different nitrate and ammonium availability in the medium; its expression increased in response to nitrate addition to the nitrate-deficient growth medium. LeNRT1-2 was detected predominantly in root hairs of trichoblasts after nitrate addition [58]. The *MdNRT1.1* gene, homologous to AtNPF6.3, was identified in the apple tree *Malus domestica* [59]. It was expressed in all organs, but the highest expression level was observed in roots. The transcription of *MdNRT1.1* was induced in response to nitrate addition to a nitrate-poor growth medium, drought, salinity, and ABA treatment. Overexpression of *MdNRT1.1* in *A. thaliana* plants resulted in increased nitrate reductase activity, total N content, main root length, number of lateral roots, and biomass production [59]. Three members of the NPF6 clade were found in the monocotyledonous plant of Lemnaceae family *Spirodela polyrhiza* (duckweed), characterized by high tolerance to abiotic stresses and used in phytoremediation [33]. The level of *SpNPF6.3* transcript in duckweed was two orders of magnitude higher than that of *AtNPF6.3* in *Arabidopsis*. Heterologous expression of *SpNPF6.3* in the *Arabidopsis chl1-5* knockout mutants rescued plant sensitivity to the toxic nitrate analog ClO_3_^−^. The results of an expression study of the *SpNRT6.3* gene and complementation analyses of the *Arabidopsis* mutants expressing *SpNRT6.3* suggested a pivotal role for the encoded protein in nitrate uptake, as well in signaling [33].

Nitrate transporters of the NPF6 family from several highly salt tolerant plants were cloned and their nitrate transport activity was demonstrated in heterologous expression systems. An *AtNPF6.3* homologue named *SaNPF6.3* was cloned from the euhalophyte *Suaeda altissima*, and the involvement of the SaNPF6.3 protein in nitrate transport was demonstrated [38]. The euhalophytes of genus *Suaeda* are unique in their ability to grow and proliferate at salinities up to 1 M NaCl [73,74,75,76]. The expression of *SaNPF6.3* in *Δynt1*, a knockout mutant of the gene for the only nitrate transporter in the methylotrophic yeast *Hansenula* (*Ogataea*) *polymorpha*, rescued the growth and nitrate transport function that were absent in the mutant [38]. The *SaNPF6.3* gene was expressed at different stages of plant development in all organs: roots, stems, leaves, and flowers. The highest level of *SaNPF6.3* expression was observed in roots and stems, which suggests the participation of this transporter in the uptake of nitrate by roots and its translocation to shoots. Similar to *AtNPF6.3* expression, that of *SaNPF6.3* was lowered in the absence of nitrate in the medium and induced by added nitrate [38]. The transcripts of four transporters homologous to AtNPF6.3, SsNRT1.1A-SsNRT1.1D, were found also in the transcriptome of the euhalophyte *S. salsa* [60]. *SsNRT1.1A* was expressed predominantly in roots, *SsNRT1.1B* in stems, and the *SsNRT1.1C* and *SsNRT1.1D* genes in leaves. The transcript levels of the *SsNRT1.1A-SsNRT1.1D* genes increased in leaves within 24 h after NaCl (400 mM) addition to the medium, with the greatest increase observed in the expression of the *SsNRT1.1C* gene. A study of the intracellular localization of the SsNRT1.1A-SsNRT1.1D proteins fused to GFP in *Arabidopsis* protoplasts revealed that, in contrast to AtNPF6.3 homologs from other plant species, SsNRT1.1A-SsNRT1.1D proteins are localized in the ER membranes. It should be noted that the presence of SsNRT1.1A-SsNRT1.1D fused with GFP in ER membranes may reflect the mislocalization of these proteins under conditions of ectopic expression in Arabidopsis protoplasts and be a result of the inhibition of the secretory pathway from the ER to PM. *A. thaliana* plants overexpressing the *SsNRT1.1A-SsNRT1.1D* genes were characterized by higher biomass production compared to wild-type plants under control conditions and greater salt tolerance under conditions of steady-state salinity for 10–15 days [60]. Nitrate-transporting activity was shown for the protein EhNPF6.3 from the halophyte *E. halophilum*. This highly salt-tolerant plant species is taxonomically, genetically, and morphologically close to *A. thaliana* [77,78]. Three genes encoding proteins of the NPF6 clade, *EhNPF6.2*, *EhNPF6.3*, and *EhNPF6.4*, were identified in the genome of the halophyte *E. halophilum*. The coding sequence of *EhNPF6.3* was cloned [39]. The amino acid sequence of EhNPF6.3 demonstrated high similarity to that of AtNPF6.3; all their key amino acid residues and motifs required for ion binding and transport function are identical, although EhNPF6.3 and AtNPF6.3 exhibited differences in the amino acid sequences in the hydrophilic cytoplasmic loop that contains binding sites for regulatory proteins (protein docking sites) [39]. The EhNPF6.3 protein demonstrated nitrate-transporting activity. The expression of *EhNPF6.3* in *Δynt1*, a knockout mutant of the gene for the sole nitrate transporter in the methylotrophic yeast *Hansenula* (*Ogataea*) *polymorpha*, restored the growth and nitrate transport that were absent in the mutant. The mutant yeast cells transformed with *EhNPF6.3* took up nitrate from the media with 200 µM and 1 mM concentrations of this anion [39]. A homolog of AtNPF6.3, a putative double-affinity nitrate transporter ZosmaNPF6.3, was recently identified in the seagrass *Z. marina*, which inhabits seawater characterized by high salinity (0.5 M), an alkaline pH (8.3), and nitrate deficiency (0–8 μM) [7]. Using the expression system of the yeast *Hansenula polymorpha*, ZosmaNPF6.3 was shown to carry out NO_3_^−^/H^+^ symport. Nitrate transport by the carrier was inhibited by alkaline pH values in the medium. The affinity of ZosmaNPF6.3 for nitrate is lower (Km = 11 μM) than that of AtNPF6.3 (Km = 50–80 μM), which may suggest the involvement of this transporter in nitrate transport at very low concentrations of this anion in the environment [7].

**Figure 3:**
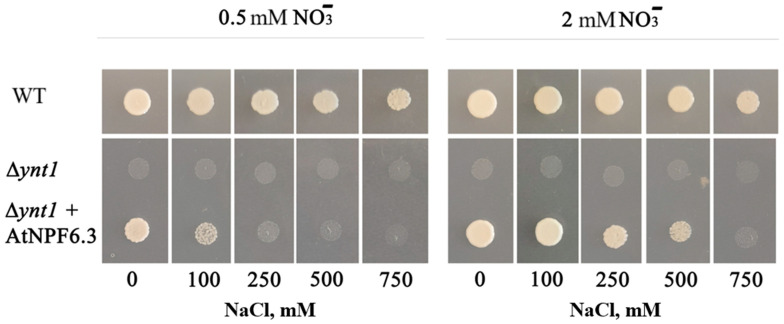
Growth of yeast *Hansenula polymorpha* (DL-1) strains, including wild-type and a knockout mutant *Δynt 1* expressing *AtNPF6.3* (*Δynt1* + *AtNPF6.3*). The yeast was grown on selective medium (SD) containing nitrate as the sole nitrogen source (0.5 mM or 2 mM KNO_3_) and NaCl at concentrations ranging from 0 mM to 750 mM NaCl. A positive control—WT *H. polymorpha* strain; negative control—*Δynt1*, a knockout mutant for the *YNT1* gene, the only nitrate transporter gene in this organism. The figure is based on the published results of the authors [38].

Chloride transport activity was shown and explored for several NPF6 proteins. This activity is interesting since it could be important for osmoregulation and Cl^−^ may compete with nitrate transport activity with essential physiological and functional consequences. Chloride-transporting activity was revealed for the AtNPF6.3 protein with low nitrate concentrations in the medium. Chloride absorption by *Xenopus* oocytes expressing *AtNPF6.3* was inhibited in the presence of nitrate [6,42]. When the conformational mobility of AtNPF6.3 was analyzed by the fluorescence resonance energy transfer (FRET) method, no significant changes in the AtNPF6.3 protein conformation were detected during chloride transport [79]. There was also no detectable binding of chloride to AtNPF6.3, as indicated by the absence of chloride ions in the crystal structure of this protein, even though crystallization was carried out in chloride-containing medium [44]. Our original results with the heterologous expression of *AtNPF6.3* in yeast *Hansenula polymorpha* indicate potential competition between nitrate and chloride with a high chloride concentration in the medium (Figure 3, based on our published results [38]). The phenomenon is potentially of high interest for nitrogen nutrition of plants under soil salinization and for halophytes.

The nitrate and chloride transport activities of AtNPF6.3 homologues from maize (*Zea mays*) have been investigated [6]. Two AtNRT1.1 homologues differing in substrate specificity, the nitrate-selective transporter ZmNPF6.6 (ZmNRT1.1B) and the chloride-selective transporter ZmNPF6.4 (ZmNRT1.1A), were cloned and studied using the *Xenopus* oocyte expression system and electrophysiology, as well as ^36^Cl^−^ and ^15^N flux measurements [6]. Both transporters are localized in the plasma membrane and transport nitrate and chloride ions in symport with protons. ZmNPF6.6 was shown to mediate high-affinity transport of nitrate; the rate of nitrate uptake as a function of the nitrate concentration exhibited a monophasic profile with saturation and Km = 210 μM. A histidine residue was found in the substrate-binding site in ZmNPF6.6 at position 362; the point mutation His362Tyr resulted in the cessation of transport of both anions. The ZmNPF6.4 protein was identified as a high-affinity chloride transporter with Km = 390 μM, and it contains a tyrosine residue in the substrate-binding site (Tyr370) instead of histidine. The Tyr370His substitution led to a change in selectivity, the start of saturation in high-affinity nitrate uptake (Km = 87 μM), and linear kinetics of chloride uptake [6]. The transport activities of ZmNPF6.4 and ZmNPF6.6 were altered by the phosphorylation/dephosphorylation of Thr106 and Thr104, respectively, but the mechanism of activity regulation differs from that of AtNPF6.3 [6]. The transport activity of these proteins was analyzed also under conditions of Cl^−^and NO_3_^−^ co-presence in the medium. An increase in the external Cl^−^ concentration had little effect on NO_3_^−^ uptake by ZmNPF6.6, but suppressed NO_3_^−^ uptake performed by ZmNPF6.4. Conversely, increasing the NO_3_^−^concentrations in the medium had no effect on Cl^−^ uptake by the ZmNPF6.4 transporter while significantly inhibiting Cl^−^ uptake accomplished by ZmNPF6.6 [6].

Chloride and nitrate transport were similarly studied for three AtNPF6.3 homologues named MtNPF6.5/MtNRT1.1A, MtNPF6.7/MtNRT1.1B, and MtNPF6.6/MtNRT1.1C from the model legume *Medicago truncatula* [29,30]. The MtNPF6.5 and MtNPF6.7 proteins are involved in both nitrate and chloride transport; oocytes expressing *MtNPF6.7* took up ^15^NO_3_^−^ at external nitrate concentrations corresponding to both high-affinity and low-affinity concentration ranges, 200 μM and 10 mM, respectively, while oocytes expressing *MtNPF6.5* transported nitrate only from the medium with 10 mM NO_3_^−^ [30]. Nitrate transport depended on the presence of a proton gradient across the membrane. The MtNPF6.6 protein had no nitrate transport activity at both concentrations and exhibited only negligible Cl^−^-transport activity. An analysis of ^36^Cl^−^ uptake showed that MtNPF6.5 and MtNPF6.7 were able to transport Cl^−^ when the concentration of this anion in the medium was about 1 mM. Nitrate in the millimolar concentration range markedly inhibited ^36^Cl^−^ uptake by the oocytes expressing *MtNPF6.5* and *MtNPF6.7*, which indicates a competitive relationship between these two anions for binding sites in both transporters. A study of knockout *mtnpf6.5-1* and *mtnpf6.5-3* mutants, ion content measurements. and fluorescence X-ray microanalysis combined with an analysis of expression patterns indicated that MtNPF6.5 was more specific for chloride, while MtNPF6.7 was more specific for nitrate [30]. Similar to AtNPF6.3, the MtNPF6.5 and MtNPF6.7 proteins have a Thr residue at position 101, which probably also plays an important role in the regulation of nitrate transport, as the point substitutions Thr101Ala and Thr101Asp completely suppressed nitrate transport by MtNPF6.5, and the Thr101Ala substitution markedly reduced nitrate transport by the MtNPF6.7 protein in the presence of different concentrations of NO_3_^−^ [30]. In addition to these AtNPF6.3 homologues, one more member of the NPF6 clade, MtNPF6.8/MtNRT1.3, is localized in the plasma membrane, is similar to the low-affinity nitrate transporter AtNRT1.3 in its amino acid sequence, and has been characterized in *M. truncatula*. It demonstrates the features of a dual-affinity nitrate transporter with biphasic high-affinity and low-affinity nitrate uptake saturation kinetics (Km = 41.6 μM; Km = 7.2 mM) [56].

Based on a sequence analysis, Xiao with coworkers [30] suggested that there are from one to four transporters homologous to AtNPF6.3 in the dicotyledonous and monocotyledonous plants, which differ in substrate binding and key regulatory amino acid residues. The authors found three haplotypes: the A-haplotype TYTF (chloride-selective), named after the key amino acid residues Thr101, Tyr356, Thr360, and Phe511 in AtNPF6.3; the B-haplotype THTF (nitrate-selective), the members of which are the functional homologs of AtNPF6.3; and the C-haplotype NQMF resulting probably from duplication of the progenitor gene and being characteristic of some proteins from legumes, in particular, alfalfa (*Medicago truncatula*), pea (*Pisum sativum*), and red clover (*Trifolium pratense*). The molecular phylogenetic analysis deduced that the B-type nitrate-selective lineage evolved from the earlier chloride-selective MtNPF6.5-like protein [30].

### 3.2. Transport of Amino Acids, Plant Hormones, and Glycosinolates

Similar to their bacterial and animal proton-coupled nutrient transporters, the protein members of the plant NPF6 clade display a diverse range of transported substrates, including amino acids, plant hormones, and glycosinolates. The membrane transporter BnNRT1.2/BnNRT1B isolated from *Brassica napus* demonstrated histidine transport in the *Xenopus* oocyte expression system [57]. Histidine transport was stimulated by alkaline pH values. Based on the kinetic analysis of histidine transport, the authors suggested a cotransport mechanism of protons and the neutral form of the amino acid. Apart from histidine, BnNRT1.2 also carried arginine and lysine, with conductance decreasing in the His > Arg > Lys series; the Km for histidine depended on the membrane potential and decreased from 25 mM at −100 mV to 1.4 mM at −180 mV [57]. For rice OsNPF6.5, the H^+^ gradient-dependent transport of selenomethionine (SeMet) has been demonstrated using yeast and *Xenopus* oocytes expression systems [80]. In *osnrt1.1b* knockout mutants, root uptake and the root-to-shoot translocation of SeMet were reduced. Overexpression of *OsNPF6.5* resulted in the opposite phenotypic traits, stimulation of SeMet uptake and transport to shoots, which promoted the accumulation of the trace element selenium in plants. This effect of *OsNPF6.5* overexpression is suggested for use in biofortification to produce plants with an elevated level of selenium in seeds [80]. The rice nitrate transporter OsNPF6.5 was linked to the absorption and assimilation of ammonium along with nitrate, which influenced the rice root microbiome [62,80,81].

AtNPF6.3 from *A. thaliana* has auxin transport activity in a heterologous expression system [82,83]. It was suggested that this property is present in plants and determines the involvement of the AtNPF6.3 protein in lateral root development, which is influenced by the soil nitrate concentration. When the concentration of nitrate in the medium is low, AtNPF6.3 exhibits auxin-transporting activity, allowing auxin efflux from cells of the apices of lateral roots: the auxin concentration in the apex decreases, which inhibits root elongation [82]. With increasing nitrate concentrations in soil, the auxin flux mediated by AtNPF6.3 decreases, which results in auxin accumulation in the tip of the lateral root, stimulating its elongation. Phosphorylation of the T101 residue in AtNPF6.3 was found to be required for auxin transport under nitrate deficiency in the medium [84]. The roles of NPF6 transporters in specific regulated fluxes of plant hormones and formation of their distribution patterns should be, however, assessed and proven individually with special care and quantitative estimates since well-studied transporters for each plant hormone are known, e.g., PIN transporters for polar auxin efflux (e.g., reviewed in [85]). MtNPF6.8 from *Medicago truncatula* was used in experiments with labelled abscisic acid ([^3^H]ABA) and enhanced the translocation of the hormone in heterologous expression systems of frog oocytes. The *MtNPF6.8* gene was expressed in all plant organs at different growth stages, but its expression was highest in shoots and the transcript level increased during growth [28,54,56,86]. The transport of glycosinolates and functional roles of the process in plants are under investigation (e.g., reviewed in [41]).

## 4. Signaling Role of NPF6 Members in the Plant Response to Nitrate and Regulatory Networks of Plant NPF6 Transporters at the Cellular Level

The functions of the NPF6 family members are not limited to the transport of nitrate and a number of other substrates. Initially, AtNPF6.3 was shown to function as a type of nitrate receptor, although not studied in the detail achieved for animal hormones. AtNPF6.3 triggers a signaling cascade that controls the expression of nitrate-regulated genes. For this binary role in both transport and signaling, it is termed a transceptor. Genes, the expression of which are controlled by nitrate, include nitrate transporters, nitrate assimilation enzymes, and those controlling root system architecture. Depending on the plant response time, two types of nitrate effects are distinguished: rapid effects that occur within minutes or hours (primary response to nitrate) and slow effects that occur over several days (secondary or long-term response to nitrate) [87,88,89].

**Figure 4:**
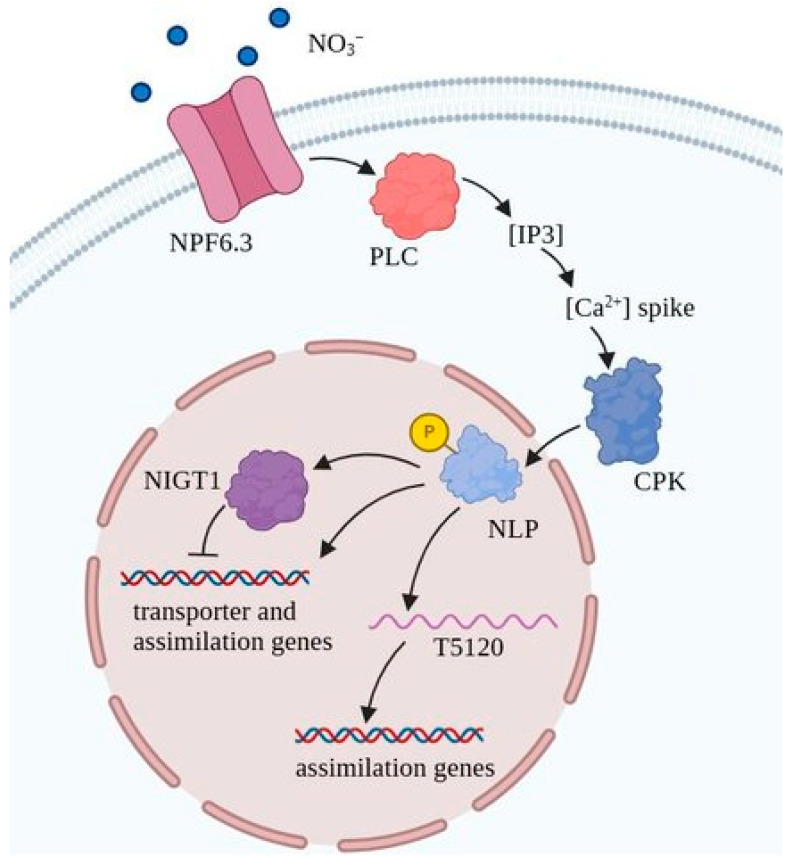
Signaling cascades triggered by AtNPF6.3 in *A. thaliana*. Nitrate binding to AtNPF6.3 results in an upregulation of phospholipase C (PLC) activity and the formation of inositol-1,4,5-triphosphate (IP3), which finally leads to a transiently increased level of cytosolic calcium. Activated calcium-dependent protein kinases (CPKs) carry out the phosphorylation of NLP transcription factors. In the phosphorylated state, NLPs translocate to the nucleus, where they trigger the transcription of the primary nitrate response genes by engaging the long non-coding RNA T5120. NLPs also activate the expression of the NIGT1 transcription factor responsible for repression of the nitrate primary response genes (based on [102]).

In the primary response to nitrate in *A. thaliana*, the transcription factors NLP6 and NLP7 (NIN-LIKE PROTEIN 6/7) are the key players: they bind to the promoters of primary nitrate response genes, altering their expression levels [90,91,92]. The sequence of events leading to the activation of NLP6 and NLP7 after nitrate binding includes a step in which the cytosolic concentration of Ca^2+^ is increased [93]. This in turn results in the activation of the Ca^2+^-dependent protein kinases CPK10/30/32. In particular, the phosphorylation of S205 in NLP7 occurs, resulting in the transfer of this protein to the nucleus and triggering the plant primary response to nitrate [94]. A transient increase in the cytosolic calcium concentration was associated with AtNPF6.3 activity, as such changes in cytosolic calcium concentrations were not observed in a series of *Atnpf6.3* knockout mutants [93,95]. Nitrate-dependent Ca^2+^-signaling also requires phospholipase C activity and an increase in the concentration of inositol-1,4,5-triphosphate in the cytosol [93]. Apart from the NLP6 and NLP7 proteins, the long non-coding RNA T5120 plays an important role in the primary response to nitrate. Although the exact mechanism underlying the role of T5120 is unknown, the transcription of this RNA was shown to be activated through direct involvement of NLP7. The latter is required for the induction of the nitrate assimilation genes but not the nitrate transporter genes [96] (Figure 4).

During the secondary response to nitrate, changes in gene expression occur more slowly than in the primary response. Many of the target genes are common to the primary and secondary responses to nitrate, but are regulated in opposite ways. For example, the expression of the high-affinity nitrate transporter gene *NRT2.1* is rapidly induced in the primary response to nitrate but is subsequently repressed in the course of the secondary response [97]. The first transcription factor shown to be involved in the secondary response to nitrate was LBD39, which carries out the repression of many nitrate-inducible genes, including NRT2.1 [98]. Four additional transcription factors, NIGT1.1/1.2/1.3/1.4, were later identified as key regulators of nitrate-inducible gene expression [99]. The expression of two of them, NIGT1.1 and NIGT 1.2, is induced both in the presence of nitrate, by the NLP7-dependent pathway, and of the reduced nitrogen forms. The other two genes (NIGT1.3 and 1.4) are expressed only in the presence of nitrate [100,101]. This indicates the existence of two negative regulatory modules involved in the NLP-NIGT1 cascade. The first is responsible for NO_3_^−^ signaling alone and involves the repressors of NIGT1.3 and NIGT1.4, whereas the second involves NIGT1.1 and NIGT1.2 and mediates a more general mechanism of response to different forms of nitrogen (Figure 4).

The network of nitrate-sensing events at the molecular level in rice has also been deciphered. Similar to AtNPF6.3 in *A. thaliana*, rice OsNPF6.3 is involved in the perception of the intracellular N status, signaling, and induction of the expression of genes responsible for nitrate and ammonium transport and assimilation (*NPF6.3B*, *NRT2.1*, *NRT2.3a*, *NIA1*, *NIR1*, *AMT1.1*, *GS1.2*, and *GOGAT1*), as well as genes regulating the transition to flowering (*Hd3a*, *Ehd1*, and *RFT1*) [61]. OsNPF6.3 was shown to induce the transfer of the NLP3 and NLP4 transcription factors into the nucleus, but other participants in this signaling pathway are not known [61]. Overexpression of *OsNPF6.3* in different rice varieties significantly accelerated flowering, shortened the time for seed maturation, and increased grain yield and nitrogen use efficiency (NUE) [61]. Conversely, a decrease in *OsNPF6.3* expression reduced nitrate and ammonium uptake by roots and lowered the expression levels of genes responsible for nitrate and ammonium assimilation and genes regulating the flowering period, which resulted in later flowering and decreased plant height, panicle size, and seed formation rate—in general lowering the yield. Overexpression of *OsNPF6.3* in *A. thaliana* resulted in an a greater increase in NUE than in rice; the plant size and biomass increased almost 2-fold and the flowering period was shortened by 6–15 days [61].

Rice OsNPF6.5 (NRT1.1B) is localized in the plasma membrane and has a similar function to AtNPF6.3. The expression of *OsNPF6.5* is stimulated by nitrate and OsNPF6.5 participates in the primary plant response to nitrate by inducing expression of the primary response genes *NRT2.1*, *NIA1*, and *NIA2* [61,62,72]. Hu et al. (2015) [62] showed that a single nucleotide polymorphism in *OsNPF6.5* of two rice varieties, *var. indica* (980 C) and *var. japonica* (980 T), which resulted in the amino acid substitution in cytoplasmic loop of the transporter, *var. indica* (Met327) and *var. japonica* (Thr327), is responsible for a higher NUE in *var. indica* than in *var. japonica*. Introgression of the *var. indica* gene into that of *var. japonica* increased nitrate uptake, nitrate root-to-shoot translocation, and the expression of genes responsible for nitrate assimilation, *NIA1* and *NIA2*. The chlorophyll content, photosynthesis rate, number of tillers, grain yield per plant, and plant biomass production were increased; in general, yield and NUE were enhanced [62].

**Figure 5:**
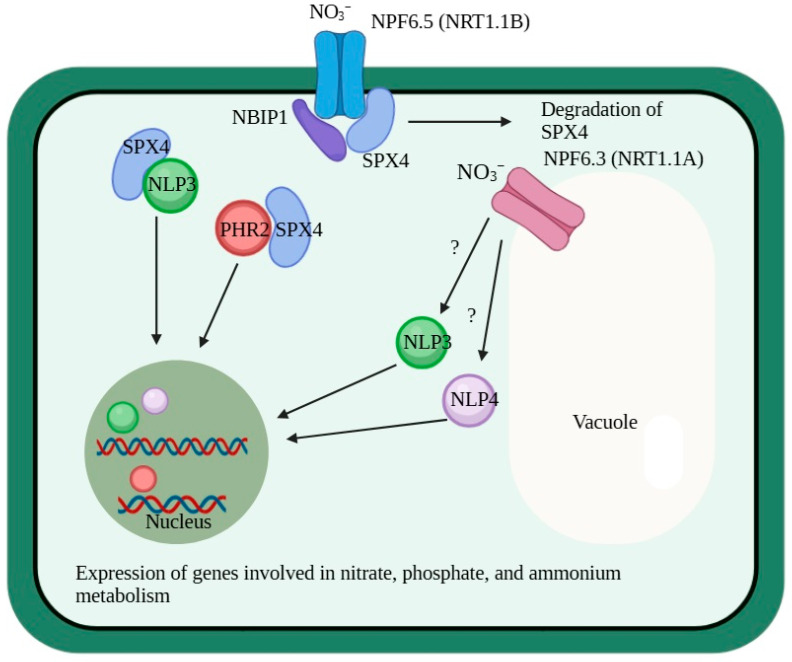
Scheme of the interactions and physiological functions of the OsNPF6.3 (NRT1.1A) and OsNPF6.5 (NRT1.1B) transceptors in *Oryza sativa*. The perception by OsNPF6.3 (NRT1.1A) and OsNPF6.5 (NRT1.1B) of nitrate in the soil solution and cytosol and transmission of nitrate signals to the nucleus involving the transcription factors NLP3 and NLP4. In the presence of nitrate in the environment, OsNRT1.1B interacts with the repressor protein OsSPX4, initiating its degradation with the participation of ubiquitin ligase NBIP1. The degradation of OsSPX4 releases the related transcription factors OsNLP3 and OsPHR2, which move into the nucleus and activate expression of genes responsible for nitrate and phosphate metabolism. Signal transduction altering the expression of genes involved in the metabolism of nitrate and ammonium is mediated by the transcription factors NLP3 and NLP4, although the other proteins in the signaling chains are not known (question mark).

A regulatory module including the OsNPF6.5-OsSPX4-OsNLP3 proteins was found to be involved in nitrate signaling from the plasma membrane to nucleus [72,103,104]. In the presence of nitrate in the medium, OsNPF6.5 interacts with OsSPX4, a repressor protein involved in phosphate signaling, leading to the degradation of the latter with the participation of NBIP1 (OsNRT1.1B Interacting Protein 1), an E3 RING-type ubiquitin ligase [103]. The OsSPX4 repressor, in turn, binds to a functional ortholog of AtNLP7, OsNLP3, which is a major transcription factor for nitrate signaling in rice and shuttles between the cytoplasm and nucleus during signal transduction. The degradation of OsSPX4 leads to the release of OsNLP3, relocation of this protein to the nucleus, and expression induction of the genes responsible for nitrogen metabolism [104]. The OsNPF6.5-OsSPX4 regulatory module enables the coordination of nitrate signaling and activation of genes involved in phosphate transport and metabolism, since the degradation of OsSPX4 in the presence of nitrate also leads to the release of the phosphate signaling transcription factor OsPHR2, which is translocated to nucleus, inducing the expression of genes responsible for phosphate transport and metabolism [72,104,105] (Figure 5).

The presence of two NPF6 forms in the rice genome may contribute to the plant adaptation to varying nitrate and ammonium availability in flood-affected land. It was suggested that OsNPF6.5 is responsible for the perception of the external nitrate signal, whereas OsNPF6.3 localized in the tonoplast perceives the intracellular nitrate concentration for the fine regulation of nitrogen utilization processes [72]. The genes encoding these proteins may be candidates for genetic manipulation to improve the yields of rice and other crops.

**Figure 6:**
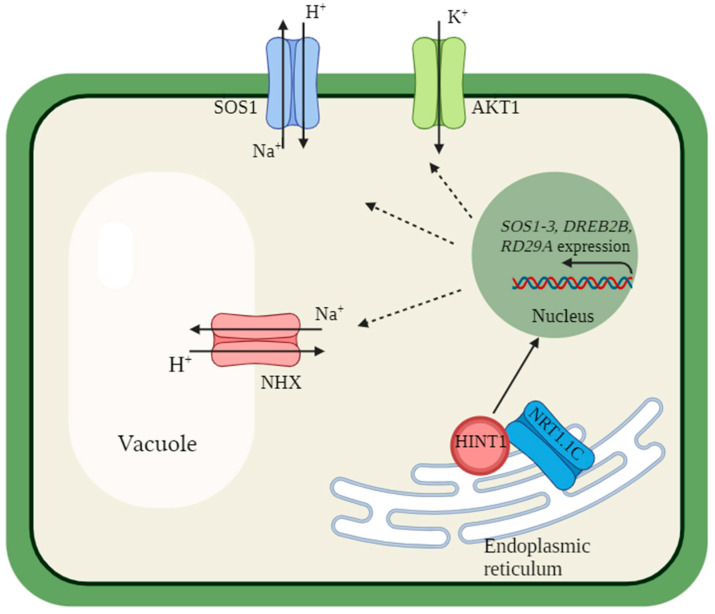
Scheme of the possible involvement of the transceptor SsNRT1.1C and the transcription factor SsHINT1 in the activation of gene expression of the SOS1–SOS3 cascade of proteins, transcription factor DREB2B, and Lea-type defense protein RD29A under salt stress according to [60]. Under conditions of salt stress, the SsNRT1.1C transceptor, which is localized in ER, initiates the relocation of the transcription factor SsHINT1 into the nucleus, which induces the expression of the SOS1–SOS3, DREB2B, and RD29A genes, resulting in Na^+^ export from cytoplasm by the PM-localized SOS1 or the tonoplast-localized NHX-type Na^+^/H^+^- antiporters.

An interesting study was completed with four transporters homologous to AtNPF6.3, SsNRT1.1A-SsNRT1.1D, found in the transcriptome of the euhalophyte *S. salsa* [60]. With the aid of the yeast two-hybrid system (Y2H) and bimolecular fluorescence complementation (BiFC), the SsNRT1.1C transporter was shown to interact with the transcription factor SsHINT1 (Histidine Triad Nucleotide-Binding Protein 1), which is homologous to AtHINT1. Overexpression of the *SsHINT1* gene in *Arabidopsis* plants induced expression of the *AtSOS1-3* genes, the genes encoding the AtDREB2B transcription factor and AtRD29A promoter, and increased the K^+^/Na^+^ ratio and plant salt tolerance. SsNRT1.1C and SsHINT1 were suggested to interact with each other in the endoplasmic reticulum under saline stress conditions [60]. SsNRT1.1C acts as a ‘sensor’ initiating SsHINT1movement into the nucleus, which activates the expression of the SOS, DREB2B, and RD29A genes (Figure 6). The transport functions of SsNRT1.1A-SsNRT1.1D proteins remain unexplored so far, and validation of their localization in the ER using marker proteins of this compartment is also required.

## 5. Roles of NPF6 Transporters in Plant Responses to Abiotic Stresses

Plant NPF6 transporters participate in responses of plants to abiotic stresses. This brief overview selects a few observations and studies, mostly with model plants, to attract attention to the roles of NPF6 transporters in stress reactions and the development of resistance. A detailed description is beyond the scope of this chapter, with more discoveries and practical applications expected in the future.

### 5.1. Salinity and Drought Stresses

The involvement of NPF6 clade of transporters in the mechanisms underlying salinity and drought tolerance is realized mainly via processes of osmoregulation and nitrogen nutrition of plants that depend on chloride and nitrate in the environment and the balance between them for osmoregulation and nitrogen nutrition. These links have not yet been properly deciphered due to the multitude of implicated players, such as cation and anion channels and transporters, plasma membrane and vacuolar H^+^-ATPases, and complex interactions between them [42,106,107].

The AtNPF6.3 protein is expressed not only in root epidermis and vasculature but also in guard cells that are directly involved in the regulation of stomatal conductance and thus in the maintenance of the ionic and water balance in plant. Mutants of *A. thaliana* for the *AtNPF6.3* gene were found to have a smaller stomatal aperture and be more drought tolerant than wild-type plants [71]. The decrease in AtNPF6.3 transport activity under drought conditions might reduce anion influx into the guard cells and prevent the depolarization of the guard cell plasma membrane, because AtNPF6.3-mediated nitrate transport is electrogenic. This would inhibit the mechanism of stomatal opening, reduce the stomatal aperture, and ultimately promote drought tolerance. There is a relationship between the negative regulation of AtNPF6.3 and ABA signaling under drought conditions. This relationship appears to occur through the protein kinases SnRK2 and CIPK23, which have been shown to be involved in both ABA signal transduction and the regulation of AtNPF6.3 activity [108,109].

Mutants of *A. thaliana* in *AtNPF6.3* demonstrated greater tolerance to high concentrations of NaCl than wild-type plants [50,110]. However, the role of AtNPF6.3 in tolerance to excess NaCl strongly depends on whether the nitrate or ammonium form of mineral nitrogen is available to plants. If nitrate was the only nitrogen source under saline stress conditions, the mutants accumulated significantly less sodium ions than wild-type plants [110]. Thus, the absence of AtNPF6.3 in the mutants is responsible for the reduced content of Na^+^, which most likely plays the role of a counterion for nitrate. When ammonium was the only source of nitrogen, the NaCl hypersensitive effect was exhibited by wild-type plants due to the excessive accumulation of chloride ions [50]. The ability of AtNPF6.3 to transport chloride [6] and the fact that *Atnpf6.3* mutants accumulated less chloride than the wild-type plants when grown on the ammonium medium [50] suggests that AtNPF6.3 is responsible for chloride uptake and accumulation under these conditions. Thus, the above studies show that the toxic effect of NaCl is due to either sodium cations or chloride anions, depending on whether nitrate or ammonium is used by the plant as the nitrogen source, respectively. The complex interactions appeared when the *MdNRT1.1* gene cloned from the apple tree *Malus domestica* was overexpressed in *A. thaliana* plants [59]: it resulted in increased nitrate reductase activity, total N content, main root length, number of lateral roots, and biomass production but the transgenic plants had reduced drought and salinity tolerance. Under these conditions, the levels of reactive oxygen species (ROS) and malondialdehyde (MDA) production, as well as the outflow of electrolytes from cells, were increased in the transgenic plants [59].

Original research of the authors with halophytes, and *Suaeda altissima* in particular, revealed changes in relative transcript level of *SaNPF6.3* in *S. altissima* under salinity stress: *SaNPF6.3* expression increased in response to NaCl shock, as well as under combined conditions of long-term salinity and low nitrate availability in the medium, suggesting an important role for this transporter in nitrogen nutrition of *S. altissima*. In contrast, the expression of *SaNPF6.3* decreased with sufficient nitrate availability (Figure 7 in [38]).

Similarly, the transcript levels of the *SsNRT1.1A-SsNRT1.1D* genes from *Suaeda salsa* increased in leaves within 24 h after NaCl (400 mM) addition to the medium, with the expression of *SsNRT1.1C* increasing more than the other genes. A study of the intracellular localization of the SsNRT1.1A-SsNRT1.1D proteins fused to GFP in *Arabidopsis* protoplasts revealed that in contrast to AtNPF6.3 homologs from other plant species, SsNRT1.1A-SsNRT1.1D proteins are localized in the ER membranes. *A. thaliana* plants overexpressing the *SsNRT1.1A-SsNRT1.1D* genes showed higher biomass production compared to wild-type plants under control conditions and greater salt tolerance under conditions of steady-state salinity for 10–15 days [60].

Data for the involvement of NPF6 clade members in plant stress responses and processes underlying stress tolerance in plants other than Arabidopsis, especially in monocotyledons, are scarce, although whole-genome sequencing of economically important monocotyledonous crops such as wheat, rice, maize, and barley should encourage such studies. The identification of the wheat NPF gene family and analysis of effects of abiotic stresses on *NPF* expression using RNA-seq and quantitative real time RT-PCR have been performed by Wang and coworkers [35]. The findings of this work have identified NPF6 members in *Triticum aestivum*, which are involved in responses to drought, high osmotic pressure, and some other stresses. In leaves, of the eight members of the wheat *NPF6* clade (*NPF6.1*-*NPF6.8*), the transcript abundance of three members (*NPF6.2*, *NPF6.3*, and *NPF6.5*) decreased several fold in response to drought, whereas the other two members (*NPF6.6* and *NPF6.8*) presented increased transcript levels under these conditions. Increasing the osmotic pressure in the medium with polyethylene glycol (PEG) activated the transcription of *NPF6.3*, *NPF6.5* and *NPF6.8*. Interestingly, drought and PEG had specific (opposite) effects on the expression of these genes, although both elevated the osmotic pressure in the medium. Heat shock lowered the transcript levels of *NPF6.2*, *NPF6.3*, *NPF6.6*, and *NPF6.8*, while a temperature decrease of up to 4 °C in the ambient medium suppressed the transcription of *NPF6.5* and elevated the levels of the *NPF6.6* and *NPF6.8* transcripts [35].

### 5.2. Potassium and Iron Deficiency, Heavy Metal Stress

Potassium is an essential macronutrient for plants that performs many important functions. Potassium ions are the major cations for keeping osmotic and turgor pressure; they are involved in the cation–anion balance and regulation of the activity of enzymes; and K^+^ is the main counter ion for nitrate in transport processes, including nitrate loading to the xylem (reviewed in [111,112]). The positive correlation between the transport of potassium and nitrate takes place at several levels of plant organization, from the whole organism to molecular level [106,113,114]. Among the number of molecular regulators of both K^+^ cellular membrane transport and the AtNPF6.3 transporter, it is worth pointing to the CBL9-CIPK23 complex of the calcineurin B-like calcium sensor CBL1/9 and protein kinase CIPK23. In particular, the inward rectifying potassium channel AKT1 is regulated by phosphorylation via protein kinase CIPK23 interacting with the calcium sensor CBL1/9 [115,116]. The CBL9-CIPK23 complex is also directly involved in the regulation of AtNPF6.3 activity by phosphorylation at T101 [49]. With high potassium and nitrate availability in the medium, CIPK23 is inactive and unable to phosphorylate AKT1 and AtNPF6.3. When potassium and nitrate availabilities are limited, the Ca^2+^-dependent activation of the CBL9-CIPK23 complex and protein phosphorylation occurs. After phosphorylation, potassium uptake through AKT1 becomes available [115,116] and AtNPF6.3 switches to high-affinity nitrate transport [49,117].

Iron is an essential microelement for plant growth and development [118]. The mutants of *A. thaliana* with the disrupted AtNPF6.3 gene were more resistant to iron deficiency than the wild-type plants, as judged by the less pronounced chlorosis in leaves of the mutant plants [119]. However, the total iron content in organs of the mutants and the wild-type plants did not differ [120]. In addition, double mutants for the major iron transporter *IRT1* and *AtNPF6.3* genes did not differ in the total iron content from the single mutant for the *IRT1* gene, and leaf chlorosis was less severe in the double mutant. These observations indicate that the increase in tolerance to iron deficiency in the mutant for *AtNPF6.3* is not due to a modulation of iron uptake from the medium but is rather mediated by iron remobilization from the shoot apoplast. Indeed, knockout mutants of *AtNPF6.3* gene had a lower cell wall pH, presumably due to the lack of proton uptake by cells through AtNPF6.3, resulting in greater apoplastic iron availability [120].

Environmental pollution by heavy metals has strong inhibitory effects on plant growth and developmental processes [121,122,123,124]. The effects of several heavy metals on *A. thaliana* plants correlated with the expression of *AtNPF6.3/NRT1.1* and nitrate uptake, although the molecular mechanisms behind these effects differed for different ions. Cadmium at 10 μM inhibited nitrate uptake and *AtNPF6.3* gene expression in seedlings. *A. thaliana* mutants in *AtNPF6.3* accumulated less cadmium in roots and stems than wild-type plants; these mutants demonstrated higher tolerance to the metal in the medium [125]. A similar inhibitory effect of a knockout mutation of *AtNPF6.3* was observed on the accumulation of zinc, but a concentration of 250 μM Zn^2+^ stimulated nitrate uptake 3–4 times by different root zones of wild-type plants [126]. Roots of Arabidopsis seedlings responded to stress of 300 μM Pb^2+^ by increasing the uptake of nitrate and expression of *AtNPF6.3* [127]. In contrast to the accumulation of cadmium and zinc, Pb^2+^ accumulation in *A. thaliana* mutants with a disrupted *AtNPF6.3* gene was higher than in the wild-type plants. This could be a consequence of higher lead bioavailability due to rhizosphere acidification by the roots of the mutants compared to the wild-type plants [127].

### 5.3. Stress Caused by High Ammonium Concentrations

Plants absorb ammonium and utilize this cation as a nitrogen source. However, high concentrations of ammonium above 0.1–0.5 mM are typically toxic to plants (reviewed in [128,129]). Arabidopsis knockout mutants in *AtNPF6.3* were more tolerant to high ammonium concentrations than wild-type plants [50,130]. However, this mutant phenotype was only expressed in the absence of nitrate, indicating its independence of the AtNPF6.3 transport function. These data provided a piece of evidence for the existence of another function of the AtNPF6.3 protein that is not directly related to nitrate transport, namely, a signaling function in response to nitrate exposure (see above). The signaling cascade activated by AtNPF6.3 after nitrate binding controls the expression of many genes, including genes encoding nitrate transporters and nitrate and ammonium assimilation enzymes [13,87]. Therefore, higher tolerance of the *Atnpf6.3* mutants to high ammonium concentrations in the absence of nitrate compared with wild-type plants could be related to the higher activity of the key enzymes of ammonium metabolism, such as glutamate dehydrogenase and glutamine oxoglutarate aminotransferase [131]. This suggestion is also supported by the ammonium tolerance of plants containing a P492L point mutation in the AtNPF6.3 protein. This substitution results in an impairment of the transport function of AtNPF6.3 but does not affect any signaling function [49]. The plants with the P492L point mutation did not differ from the wild-type plants in tolerance to high concentrations of ammonium, i.e., the absence of only one function of AtNPF6.3, namely, the transport function, was sufficient for exhibiting symptoms of ammonium toxicity [131]. This was not the case in the presence of nitrate in the medium. As the concentration of nitrate in the medium increased, there was an uneven increase in resistance to ammonium toxicity in the wild-type and *Atnpf6.3* knockout mutant plants [132]. Mutant plants lagged far behind wild-type plants in developing resistance, and already at a nitrate concentration of 0.2 mM, the resistance of knockout mutant and wild-type plants matched. With a further increase in the nitrate concentration, the wild-type plants became more resistant than the *Atnpf6.3* mutants, i.e., reversion of the knockout mutant phenotype occurred. A more pronounced increase in ammonium tolerance in the wild-type plants than in *Atnpf6.3* mutants has been attributed to the prevention of the excessive acidification of the medium [132]. The conclusion is that one of the toxic effects of ammonium is related to the accumulation of protons in the medium and lowering the external pH, which occurs during ammonium absorption and assimilation [132]. The wild-type plants better cope with environmental acidification in the co-presence of ammonium and nitrate due to AtNPF6.3-mediated 2H^+^/NO_3_^−^ symport. Interestingly, SLAH3, the slow anion efflux channel that forms a complex with AtNPF6.3, is also involved in this process. This complex performs cyclic nitrate transport associated with the absorption of protons from the medium and thus participates in maintaining the optimal pH in the rhizosphere [132].

## 6. Conclusions and Perspectives

In summary, proteins of the NPF6 subfamily are present in and have been studied in both dicotyledonous and monocotyledonous plant species. Typically, these are the nitrate transporters that are represented by several members (Figure 1; Table 1). Some of the plant species studied have four AtNPF6 homologues, similar to *Arabidopsis thaliana*; other plants have more different NPF6 subfamily members. These proteins carry out a number of diverse functions, with nitrate transport among the most studied and important of them. The NPF6 transporters are involved in the uptake of nitrate by roots and allocation of this anion to the different tissues and organs of the plant. Among these proteins, there are both low-affinity and high-affinity nitrate transporters, which promote the ability of plants to inhabit environments with nitrate concentrations ranging from several µM to tens of mM. Some members of the NPF6 subfamily, in particular AtNPF6.3 [24], are characterized by dual affinity for nitrate. The AtNPF6.3 transporter has been shown able to switch between the low- and high-affinity operation modes by changing the phosphorylation status of the key threonine residue Thr101 [45,65]. This feature allows quick plant responses to fluctuations in environmental nitrate concentrations before the changes in the gene expression of the other nitrate transporters start.

Members of NPF6 clade also transport chloride and are responsible for the uptake of this anion, which is present in the environment more often in relatively low concentrations but is required by plants as an essential micronutrient [133,134]. The NPF6 family members carry out both low-affinity and high-affinity chloride transport, each presumably contributing to chloride uptake from the environment. The high-affinity chloride transporter ZmNPF6.4 in maize [6] and the low-affinity MtNPF6.5 in alfalfa have been identified [30]. According to thermodynamic considerations, an energy supply is required for chloride uptake by plant cells in most cases, except for severe chloride salinization [135]. Proton-coupled pH-dependent Cl^−^ influx activity has been demonstrated for ZmNPF6.4 [6] and MtNPF6.5 [30] in an oocyte expression system and in *Sinapis alba* root hairs in electrophysiological experiments [136].

Plasma membrane localization was revealed for most of the known NPF6 proteins; this suggests a significant contribution of NPF6 subfamily members to maintaining the proper balance of nitrate and chloride fluxes across this membrane, ensuring priority in the uptake of NO_3_^−^ over Cl^−^ when the latter predominates. Such a chloride predominance occurs, in particular, under NaCl stress conditions. Maintaining the proper balance of nitrate and chloride fluxes is realized via the regulation of the expression of nitrate and chloride NPF6 transporters. The regulation of the expression of nitrate and chloride transporters with participation of the NLP transcription factors has been demonstrated (Figure 4 and Figure 5). Along with manipulations of the expression of the nitrate and chloride transporter genes, changing the key amino acid in the anion binding sites may be an approach to developing plant varieties with improved nitrate selectivity when cultured under soil nitrate deficiency and salinity.

Apart from ion transport, NPF6 proteins also play an important role in nitrate signaling. For example, the activation of signaling systems with the participation of AtNPF6.3 as a nitrate receptor leads to the induction of gene expression involved in the primary and secondary plant responses to nitrate. In particular, the expression of genes for high-affinity nitrate transporters and nitrogen assimilation is activated [87,88,89]. In the primary and secondary responses, a number of transcription factors are involved, such as NLP6 and NLP7 [90,91,92], LBD39 [98], and NIGHT1.1/1.2/1.3/1.4 [99]. In the time course of the responses, the concentration of cytosolic Ca^2+^ temporarily increases [93], which activates Ca^2+^-dependent protein kinases CPK10/30/32 [94] that phosphorylate transcription factors. All of these regulatory proteins could be targets for rewiring regulatory networks and engineering varieties with improved traits.

Proteins of the NPF6 subfamily also participate in transport of phytohormones. The multiple functionality of NPF6 proteins determines their important roles in key physiological processes, such as nitrogen nutrition and assimilation, and plant growth and development. Participation in growth and developmental processes is realized not only through nitrogen nutrition and nitrate signaling but also through the hormonal status of growing organs. NPF6 proteins also participate in plant responses to different stresses and the build-up of tolerance to stresses such as drought, soil salinity, potassium and iron deficiency, and heavy metal and ammonium toxicity. Defensive reactions involve both the transport and signaling functions of NPF6 proteins. It is evident that the multiplicity of NPF6 proteins that have evolved in various species is required for a wide range of physiological functions under constantly changing habitat conditions to overcome the impacts of stress.

The perspectives of using NPF6 subfamily members for genetic engineering/editing of economically and agriculturally important plant species are under discussion (e.g., [137]) with the essential work mentioned above and completed for rice ([62,104]; reviewed in [138]). However, more essential research is required to decipher the specific mechanisms behind the regulation and functions of these transporters. The complexity of any biological organism studied by systems biology with numerous nonlinear feedbacks and interactions may hamper the direct, simple approaches. Moreover, the regulatory aspects and ecological impacts should be considered.

## Figures and Tables

**Figure 7:**
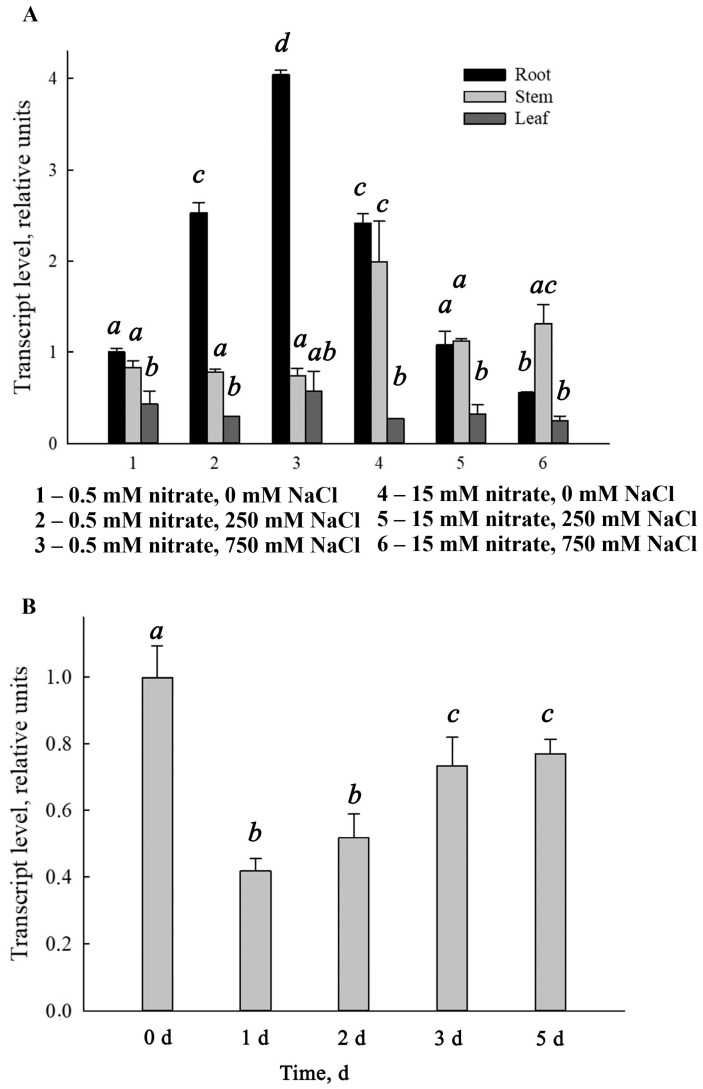
Relative abundance of *SaNPF6.3* transcripts in *S. altissima* plants. (**A**) *SaNPF6.3* expression in roots, stems and leaves with different NaCl concentrations in the nutrient solution and the background of either low (0.5 mM) or high (15 mM) nitrate availability. (**B**) Dynamics of *SaNPF6.3* expression in the roots of plants grown under conditions of high nitrate availability following the transfer of the plants to the same medium without nitrate. Data are shown as the means ± SDs from three independent experiments. Bars with different letters are significantly different at *p* < 0.05. The results were deduced from three biological replicates and each of them was performed in three analytical replicates. The figure is based on the published results of the authors [38].

**Table 1:** Representative nitrate transporters of clade 6 of the NPF/NRT1 family in the dicotyledonous (*Arabidopsis thaliana*, *Medicago truncatula*, *Brassica napus*, *Solanum lycopersicum* (*Lycopersicon esculentum*), *Malus domestica*, *Suaeda altissima*, *Eutrema halophilum*, and *Salicornia europaea*) and monocotyledonous (*Zea mays*, *Oryza sativa*, *Sorghum bicolor*, *Brachypodium distachyon*, *Zostera marina*, and *Spirodela polyrhiza*) plants.

Species Name	Gene Name, Locus *, Gene Product Localization	Haplotype According to Xiao et al., (2021) [30]	The Substrate-Binding Amino Acid Residues Determining the Transporter Haplotype	Functions and Physiological Roles	References
*Arabidopsis thaliana*	AtNPF6.3 (NRT1.1/CHL1.1), AT1G12110, plasma membrane	B	Thr101/His356/Thr360/Phe511 THTF	Dual-affinity nitrate transporter, auxin transport, nitrate signaling, chloride transport in the absence of nitrate	[4,24,45,50]
	AtNPF6.2 (NRT1.4), AT2G26690, plasma membrane	–	Thr98/Tyr346/Ile350/Phe498 TYIF	Low-affinity nitrate transporter, nitrate deposition in the leaf petiole	[51]
	AtNPF6.4 (NRT1.3), AT3G21670	–	Met103/Tyr352/Thr356/Phe502 MYTF	Nitrate transport?	[52,53]
*Medicago truncatula*	MtNPF6.5 (NRT1.1A), Medtr4g101380, plasma membrane	A	Thr101/Tyr356/Thr360/Phe515 TYTF	Nitrate and chloride transport, Cl-selective; chloride uptake by root cells	[30]
	MtNPF6.7 (NRT1.1B), Medtr5g012290, plasma membrane	B	Thr101/His354/Thr358/Phe512 THTF	Nitrate and chloride transport, NO_3_^−^–selective; participation in nodule formation	[28,30,54,55]
	MtNPF6.6 (NRT1.1C), Medtr5g012270	C	Asn103/Gln355/Met359/Phe514 NQMF	Nitrate transport, NO_3_^−^–selective?	[30]
	MtNPF6.8 (NRT1.3) (Medtr5g093170.1), plasma membrane	–	Thr104/Tyr350/Asn354/Tyr500 TYNY	Dual-affinity nitrate transporter, ABA transport	[28,54,56]
*Brassica napus*	BnNRT1.2 (NRT1B)	B	Thr100/His355/Thr359/Phe510 THTF	Low-affinity transport of nitrate and amino acids (histidine, arginine, and lysine)	[57]
*Solanum lycopersicum* (*Lycopersicon esculentum*)	LeNrt1-1	A	Thr102/Tyr356/Thr360/Phe515 TYTF	Nitrate transport? Nitrate uptake by root cells?	[58]
	LeNrt1-2	A	Thr102/Tyr353/Thr357/Phe510 TYTF		
*Malus domestica*	MdNRT1.1	A	Thr105/Tyr359/Thr363/Phe518 TYTF	Nitrate transport, nitrate signaling, and participation in abiotic stress responses	[59]
*Suaeda altissima*	SaNPF6.3 (NRT1.1)	A	Thr106/Tyr358/T363/F517 TYTF	Low-affinity nitrate transport	[38]
*Suaeda salsa*	SsNRT1.1A, endoplasmic reticulum	A-like	Thr106/Tyr358/Ser362/Phe517 TYSF	?	[60]
	SsNRT1.1B, endoplasmic reticulum	B	Thr103/His355/Thr359/Phe514 THTF	?	[60]
	SsNRT1.1C, endoplasmic reticulum	A-like	Thr98/Tyr348/Ile352/Phe502 TYIF	?	[60]
	SsNRT1.1D, endoplasmic reticulum	A	Thr103/Tyr349/Thr353/Phe498 TYTF	?	[60]
*Eutrema halophilum*	EhNPF6.3	B	Thr100/His355/Thr360/Phe510 THTF	?	[39]
*Salicornia europaea*	SeNPF6.3(NRT1.1A)	A-like	Thr114/Tyr366/Ser370/Phe525 TYSF	?	Salicornia DB (https://www.salicorniadb.org/ (accessed on 15 December 2024))
	SeNRT1.1B	B	Thr106/His360/Thr364/Phe520 THTF	?	
	SeNRT1.1C	A-like	Thr98/Tyr347/Ile351/Phe501 TYIF	?	
	SeNRT1.1D	–	Thr114/Tyr363/Ser367/Asn520 TYSN	?	
*Zea mays*	ZmNPF6.4 (NRT1.1A) (GRMZM2G086496_P01), plasma membrane	A	Thr106/Tyr370/Thr374/Phe528 TYTF	High-affinity chloride transporter, chloride uptake by root cells?	[6,25]
	ZmNPF6.6/NRT1.1B (GRMZM2G161459_P02), plasma membrane	B	Thr104/His362/Thr374/Phe528 THTF	High-affinity nitrate transporter, chloride transport in the absence of nitrate	[6,25]
	ZmNPF6.7/NRT1.1C (GRMZM2G112154_P01)	B	Thr104/His356/Thr360/Phe507 THTF	?	[25]
	ZmNPF6.5/NRT1.1D (GRMZM2G161483_P01)	A	Thr109/Tyr368/Thr372/Phe519 TYTF	?	[25]
*Oryza sativa*	OsNRT1.1A/OsNPF6.3 (LOC_Os08g05910.1), tonoplast	A	Thr107/Tyr366/Thr370/Phe523 TYTF	Dual-affinity nitrate transporter, a putative cytosolic nitrate sensor	[61]
	OsNRT1.1B/OsNPF6.5, (LOC_Os10g40600.1), plasma membrane	B	Thr104/His362/Thr366/Phe514 THTF	Dual-affinity nitrate transporter, perception of an external “nitrate” signal	[62]
	OsNRT1.1C (LOC_Os03g01290.1)	A	Thr118/Tyr369/Thr373/Phe517 TYTF	Chloride transport?	[25]
*Sorghum bicolor*	SbNRT1.1A (Sb07g003690.1)	A	Thr106/Tyr364/Thr368/Phe521 TYTF	Chloride transport?	[25]
	SbNRT1.1B (Sb01g029470.1)	B	Thr104/His359/Thr363/Phe513 THTF	Nitrate transport?	[25]
	SbNRT1.1C (Sb01g050410.1)	A	Thr113/Tyr374/Thr378/Phe525 TYTF	Chloride transport?	[25]
*Brachypodium distachyon*	BdNRT1.1A (Bradi3g16670.1)	A	Thr104/Tyr363/Thr367/Phe519 TYTF	Chloride transport?	[25]
	BdNRT1.1B (Bradi3g33030.1)	B	Thr108/His365/Thr369/Phe516 THTF	Nitrate transport?	[25]
	BdNRT1.1C (Bradi3g33040.1)	B	Thr112/His367/Thr371/Phe518 THTF	Nitrate transport?	[25]
	BdNRT1.1D (Bradi1g78330.1)	A	Thr124/Tyr381/Thr385/Phe529 TYTF	Chloride transport?	[25]
*Zostera marina*	ZosmaNPF6.3	A	Thr103/Tyr350/Thr354/Phe509 TYTF	Putative dual-affinity nitrate transporter	[7]
*Spirodela polyrhiza*	SpNPF6.3(NRT1.1A)	A	Thr102/Tyr353/Thr/Phe TYTF	Nitrate transport?	[33]

* Locus numbers were obtained from the TAIR (*A. thaliana*; https://www.arabidopsis.org/, accessed on 15 December 2024) and Phytozome (*M. truncatula*, *Z. mays*, *O. sativa*, *S. bicolor*, *B. distachyon*; https://phytozome-next.jgi.doe.gov/, accessed on 15 December 2024) databases.

## Data Availability

Data are contained within the manuscript.

## Supplementary Materials

The following supporting information can be downloaded at https://www.mdpi.com/article/10.3390/ijms252413648/s1.
