# Supplementary material for: Functional and Molecular Characterization of Plant Nitrate Transporters Belonging to NPF (NRT1/PTR) 6 Subfamily
Source: Int J Mol Sci. 2024 Dec 20;25(24):13648. doi: 10.3390/ijms252413648 (PMC11677463; doi:10.3390/ijms252413648)
Supplement: Supplementary file 1 [file ijms-25-13648-s001.zip › ijms-3345524-supplementary.pdf]

Supplementary materials

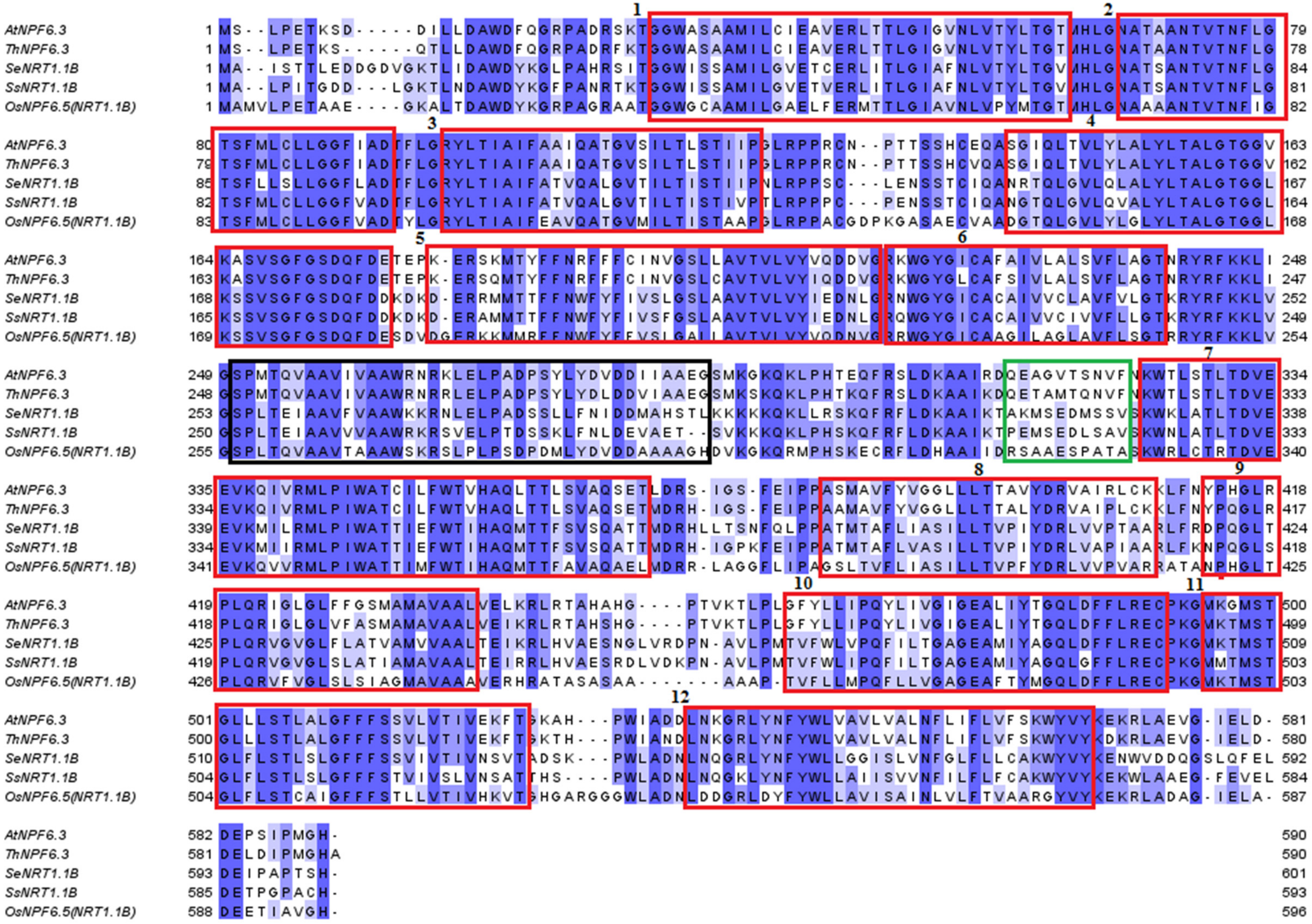

Figure S1. Amino acid sequence alignment of glycophytes (*A. thaliana*, *O. sativa*) and halophytes (*Suaeda salsa, Thellungiella halophila, Salicornia europaea*) performed in MAFFT program (https://www.ebi.ac.uk/Tools/msa/mafft/) and visualized in Jalview (https://www.jalview.org/). Red indicates transmembrane α-helices (1-12), black indicates the lateral cytoplasmic α-helix; green indicates the region located between the cytoplasmic α-helix and the 7th TM domain, which may play an important role in nitrate uptake by roots and translocation to shoots, as well contribute to the determination of NUE value.

Table S1. Amino acid sequences of proteins used for phylogenetic analysis.

Species name	Transporter name	NCBI's GenBank number
Arabidopsis thaliana	AtNPF6.1	NP_196844.1
	AtNPF6.2	NP_850084.1
	AtNPF6.3	NP_563899.1
	AtNPF6.4	NP_188804.1
Suaeda altissima	SaNPF6.3	OQ330855
Thellungiella halophila (*Eutrema salsugineum*)	ThNPF6.3	Thhalv10007157
	ThNPF6.2(A)	Thhalv10001943
	ThNPF6.2(B)	Thhalv10001953
	ThNPF6.4	Thhalv10020360
Salicornia europaea	SeNPF6.3	Seu_g3422.t1*
Spirodela polyrhiza	SpNPF6.3(NRT1.1A)	Spipo1G0085300*
Zostera marina	ZosmaNPF6.3	KMZ72028.1
Medicago truncatula	MtNPF6.4	XP_003609313.1
	MtNPF6.5(NRT1.1A)	XP_003608752.1

	MtNPF6.6 (NRT1.1C)	XP_003611284.1
	MtNPF6.7 (NRT1.1B)	XP_003608752.1
	MtNPF6.8 (NRT1.3)	GU966590.1
Sorghum bicolor	SbNRT1.1A	XM_002443834.2
	SbNRT1.1B	XM_002464914.2
	SbNRT1.1C	XM_002468647.2
Zea mays	ZmNPF6.1	NP_001170202.1
	ZmNPF6.2	XP_008675518.1
	ZmNPF6.3	NP_001169613.1
	ZmNPF6.4(NRT1.1A)	BT053880.1
	ZmNPF6.5(NRT1.1D)	XM_008651564.2
	ZmNPF6.6(NRT1.1B)	XM_008660202.3
	ZmNPF6.7(NRT1.1C)	XM_008660209.3
	ZmNPF6.8	NP_001281186.1
Oryza sativa	OsNPF6.3(NRT1.1A)	XP_015650127.1
	OsNPF6.5(NRT1.1B)	XP_015614015.1
	OsNPF6.4(NRT1.1C)	XP_015632236.1
Brachypodium distachyon	BdNRT1.1A	XP_003573480.1
	BdNRT1.1B	XP_003574312.1
	BdNRT1.1C	XP_003572060.1
	BdNRT1.1D	XP_003562233.3
Malus domestica	MdNPF6.3	XP_008358135.1

Brassica napus	BnNRT1.1B	XP_013698339.2
Lycopersicon esculentum	LeNrt1-1	XP_004245746.1
	LeNrt1-2	XP_004244572.1

Note. The numbers given for the *Spirodela polyrhiza* and *Salicornia europaea* transporters are taken from the Phytozome (https://phytozome-next.jgi.doe.gov/info/Spolyrhiza_v2) and *Salicornia* DB (https://www.salicorniadb.org/) databases, respectively.
